# Thank you to Reviewers and Editors for 2023

**DOI:** 10.1093/plcell/koae031

**Published:** 2024-02-02

Editor-in-Chief Blake C. Meyers and the staff of *The Plant Cell* thank the reviewers listed below, whose insight and contributions in reviewing manuscripts from January 1 to December 31, 2023, are crucial to the quality of the literature we publish, and to the journal’s success.

We would also like to express our deep gratitude to the Senior Editors, Reviewing Editors, Guest Editors, and Assistant Features Editors who lend their outstanding expertise and service to the journal, including several editors who began or ended their terms in 2023, and are listed below.

## REVIEWERS

Patrick Achard

Natalia Patricia Achkar

Guido Van den Ackerveken

Zach Adam

Javier Agustí

Sebestian Eves-van den Akker

Armando Albert

Isabell Albert

Pablo Albertos

Andrew Charles Allan

Randy D Allen

Juliana Almario

Jose M Alonso

Saleh Alseekh

José M Alvarez

Rubén Rellán Álvarez

Richard M. Amasino

Herbert van Amerongen

Samuel Amsbury

Carl Andre

Fernando Andrés

Alexis De Angeli

Ruthie Angelovici

Pedro J, Aphalo

Ute Armbruster

Albrecht G. von Arnim

Laura Arribas-Hernández

Mario Alberto Arteaga-Vázquez

Tadao Asami

Sylvain Aubry

Utku AVCI

Michael J Axtell

Andreas Bachmair

Pierre Baduel

Elena Baena-Gonzalez

Quinten Bafort

Sacha Baginsky

Sasha Baginsky

Ming-Yi Bai

Julia Bailey-Serres

Salma Balazadeh

Martin Balcerowicz

Janneke Balk

Steven G. Ball

Mark J Banfield

Liang Bao

Alice Barkan

Fredy Barneche

Rebecca Bart

Carlisle S. Bascom

George Bassel

Gilles Basset

Diane C. Bassham

Philip David Bates

Sébastien Baud

Martin Bayer

Jérémie Bazin

Frederic Beaudoin

Annette Becker

Claude Becker

Dirk Becker

Borja Belda-Palazon

Marian Bemer

Abdelhafid Bendahmane

Kyle W. Bender

Moussa Benhamed

Malcolm J. Bennett

Oliver Berkowitz

Christopher E Berndsen

Edoardo Bertolini

Marco Betti

Michael W Bevan

Susheel Sagar Bhat

Kaushal K Bhati

Devaki Bhaya

Rahul Bhosale

Guozhi Bi

Jenia Binenbaum

Kevin A Bird

Anthony Bishopp

Ton Bisseling

Crysten Blaby-Haas

Jantana Blanford

Robert E Blankenship

Mike R. Blatt

Miguel A. Blazquez

Arnold Jeffrey Bloom

Scott Andrew Boden

Roeland Boer

Petra C Boevink

Steven Boeynams

Jorunn Bos

Maurice Bosch

Javier Francisco Botto

Marie Boudsocq

Yohann Boutté

John L. Bowman

Tolga Osman Bozkurt

David M Braun

Peter Brodersen

Matthew D Brooks

John Browse

Scott Bruce

Javier Brumos

Guillaume Brun

Jacob O Brunkard

Qingyun Bu

Felix E. Buchert

C. Robin Buell

Tessa Burch-Smith

Marco Bürger

Ingo Burgert

Steven Burgess

Yogev Burko

Adrien Burlacot

Vincent Burlat

Katharina Bürstenbinder

Edgar B. Cahoon

Judy Callis

Damián A Cambiagno

Ana Campilho

Hui Cao

Yangrong Cao

Jeffrey L. Caplan

Warren Cardinal-McTeague

Philip Carella

Jorge Jose Casal

Baptiste Castel

Carmen Castresana

Christian Danve Marco Castroverde

Marco Catoni

Luigi Cattivelli

Jorge Rodríguez Celma

Jijie Chai

Raquel Lia Chan

Hao-Xun Chang

Thanin Chantarachot

Fabien Chardon

Dana Charuvi

Dijun Chen

Guanqun Gavin Chen

Haodong Chen

Letian Chen

Li-Yu Chen

Meng Chen

Qian Chen

Xiangsong Chen

Xiao-Ya Chen

Xuewei Chen

Zhixiang Chen

Zhonghua Chen

Xiaofei Cheng

Trinh Duy Chi

David Chiasson

Andrea Chini

Po-Lin Chiu

Jungnam Cho

Monika Chodasiewicz

Giltsu Choi

Kang Chong

Frédéric Choulet

Chengcai Chu

George Chuck

James Clark

Nuria S. Coll

Lucia Colombo

Gavin C Conant

Jose L. Crespo

Shiri Graff van Creveld

Pedro Crevillén

Peter Alexander Crisp

Roberta Croce

Quentin CB Cronk

Pilar Cubas

James N Culver

Josh Cuperus

Kirk J Czymmek

Danilo M. Daloso

Jose-Antonio Daros

Maitrayee DasGupta

Agata Daszkowska-Golec

Sourav Datta

Jean-Michel Daviere

Seth J. Davis

Tom Davis

Roger B Deal

Juan M Debernardi

Thomas A. DeFalco

Matteo Dell'Acqua

Dean DellaPenna

Taku Demura

Jurgen Denecke

Shulin Deng

Willem desmedt

Thierry M. Desnos

Ralf Georg Dietzgen

Aalt D.J. van Dijk

Shou-Wei Ding

Yiliang Ding

Zhaojun Ding

Nico Dissmeyer

Michael Anthony Djordjevic

Grazyna Dobrowolska

Antony N Dodd

Ian C Dodd

Peter N. Dodds

Peter Doermann

Vivek Dogra

Hui Dong

Juan Dong

Suomeng Dong

Yihan Dong

Daolong Dou

Georgia Drakakaki

Ludovico Dreni

Thomas Dresselhaus

Wolfgang Dröge-Laser

Jiamu Du

Qiaohong Duan

Christian Dubos

Daniel C. Ducat

Luke T Dunning

Peter J. Eastmond

Lutz Andreas Eichacker

Michael J. Emes

Benjamin Engel

Juergen E. Engelberth

Timo Engelsdorf

Mark Estelle

Peter Etchells

Holger Eubel

Costa Fabrizio

Christian Fankhauser

Karoly Fatyol

Christine Faulkner

Mitchell Feldmann

Matyas Fendrych

Feng Feng

Jian Feng

Shouqian Feng

Alisdair R. Fernie

Cristina Ferrandiz

Maria Immacolata Ferrante

Simone Ferrari

Yosef Fichman

Duarte D. Figueiredo

Iris Finkemeier

Scott A. Finlayson

Andrew J. Fleming

Stefan de Folter

Eloise Foo

John E. Fowler

Christine H. Foyer

Jessica Y Franco

Margaret Frank

Vernonica (Noni) E Franklin-Tong

Joanna Denise Friesner

Jiří Friml

Da-Qi Fu

Xiangdong Fu

Ying Fu

Zheng Qing Fu

Anja Thoe Fuglsang

Naoko Fujita

Jutarou Fukazawa

Toshiyuki Fukuhara

Hideya Fukuzawa

Cecilia Rodriguez Furlan

Selma Gago-Zachert

Kimberly L Gallagher

Sean D Gallaher

Andrea Gallavotti

Jean Luc Gallois

Caiji Gao

He Gao

Zhenyu Gao

Gyozo Garab

Juan Antonio García

Charles S. Gasser

Marina Gavilanes-Ruiz

Mary Gehring

Dietmar Geiger

Kasper van Gelderen

Niko Geldner

Joshua M Gendron

Jonathan I. Gent

Jonathan Gershenzon

Koen Geuten

Miriam L Gifford

Carmela Giglione

Matthew Gilliham

Stewart Gillmor

James J. Giovannoni

Beatrice Giuntoli

Katarzyna Glowacka

Michel Goldschmidt-Clermont

Alexander Goldshmidt

Lei Gong

Zhizhong Gong

Daniel H. Gonzalez

Nathalie Gonzalez

Cecilia Gotor

Benjamin Gourion

Aymeric Goyer

John Gray

William M Gray

Thomas Greb

Kathleen Greenham

Thomas Griebel

Arthur R. Grossman

Guido Grossmann

Ueli Grossniklaus

Marcin Grzybowski

Xiaofeng Gu

Yangnan Gu

Anthony Guihur

Fang-Qing Guo

Jingxin Guo

Liang Guo

Weilong Guo

Wen-Wu Guo

Rodrigo A Gutiérrez

Emilio Gutierrez-Beltran

Jose Gutierrez-Marcos

Caroline Gutjahr

Anders Hafrén

Barbara Ann Halkier

Takahiro Hamada

Ulrich Zeno Hammes

John P. Hammond

Fangpu Han

Zhenhai Han

Robert D Hancock

Runlai Hang

Taslima Haque

Jeremy Harbinson

Christian S. Hardtke

Alex Harkess

Jake Harris

Maria J. Harrison

Thomas Hartwig

Wendy Harwood

George W. Haughn

Angela Hay

Ken-ichiro Hayashi

Michael J Haydon

Michael Lloyd Hayes

Chuan He

Hang He

Junxian He

Xin-Jian He

Yuehui He

Kai Heimel

Karolina Heyduk

Jefri Heyman

Michael Hippler

Heribert Hirt

Kei Hiruma

Cheng Hsun Ho

Tuan-hua David Ho

Daniel Hofius

Herman Höfte

David R. Holding

Guo Hongqing

Hanna Hõrak

Shuguo Hou

Xingliang Hou

Gregg A. Howe

Stephen H. Howell

Polly Yingshan Hsu

Jianhong Hu

Jian Hua

Hongda Huang

Jirong Huang

Li-Fen Huang

Rongfeng Huang

Sanwen Huang

Shanjin Huang

Shao-shan Carol Huang

Shuai Huang

Elton Hudson

Arthur G. Hunt

Aman Husbands

Youbong Hyun

Miho Ikeda

Takehito Inaba

Marco Incarbone

Gwyneth C. Ingram

Takashi Ishida

Ryo Ishikawa

Toshiro Ito

Hiro-oki Iwakawa

Masanori Izumi

Laura Jaakola

Pierre Jacob

Yannick Jacob

Steve Jacobsen

Georg Jander

Geng-Jen Jang

Hanna Janska

Paul Jarvis

Jong-Seong Jeon

Jeeyon Jeong

Joseph M. Jez

Min Jia

Jiming Jiang

Kun Jiang

Ning Jiang

Wenbo Jiang

Jose M Jimenez-Gomez

Honglei Jin

Giles N Johnson

Matthew P Johnson

Jonathan D.G. Jones

Kevin L Cox Jr

Thomas Juenger

Pauline E Jullien

Muhammed Jamsheer K

Eirini Kaiserli

Kaisa Kajala

Nitin Uttam Kamble

Byung-Ho Kang

Stanislaw Karpinski

Farid El Kasmi

David M. Kehoe

John Kelly

Felix Kessler

Sharon A. Kessler

Tijs Ketelaar

Inna Khozin-Goldberg

Takatoshi Kiba

Daniel Kierzkowski

Tetsu Kinoshita

Daniel J. Kliebenstein

Esther van der Knaap

Volker Knoop

Yuriko Kobayashi

Karen E. Koch

Claudia Köhler

Fanjiang Kong

Craig Vander Kooi

Maarten Koornneef

Hiroyuki Koyama

David M. Kramer

Marianne C Kramer

Anja Krieger-Liszkay

Beth A. Krizek

Naden Krogan

Thomas Kroj

Joanna Kufel

Prakash P. Kumar

Khushbu Kumari

Pritha Kundu

Hans-Henning Kunz

Yukio Kurihara

Makoto Kusaba

Bernhard Kuster

Benoît Lacombe

Hon-Ming Lam

Zhaobo Lang

Jane A. Langdale

Dmitry Lapin

Emily R Larson

Sascha Laubinger

Patrick Laufs

Thomas Laux

Ji-Young Lee

Travis A Lee

Yun Sun Lee

James Leebens-Mack

Samuel Leiboff

Pablo Leivar

Patricia Leon

Amnon Lers

Avraham A. Levy

Jennifer D Lewis

Bingbing Li

Chuanyou Li

Gang Li

Hong-Ju Li

Hsou-min Li

Jia Li

Jiayang Li

Jigang Li

Jing Li

Lin Li

Mnigjiu Li

Pinghua Li

Ruixi Li

Song Li

Wen-Xue Li

Xiaobo Li

Xueyong Li

Yunhai Li

Zhengguo Li

Yan Liang

Yun-Kuan Liang

Gurrieri Libero

Honghui Lin

Jinxing Lin

Rongcheng Lin

Wen-Hui Lin

Baohui Liu

Bo Liu

Chun-Ming Liu

Junfeng Liu

Luning Liu

Qun Liu

Wende Liu

Xigang Liu

Yule Liu

Zhongsong Liu

Clive Lo

L. Maria Lois

Terri A. Long

Yuchen Long

Sara Lopez-Gomollon

Oscar Lorenzo

Tiago Filipe Lourenço

Congming Lu

Weijiang Luan

Jessica R Lucas

Peter Knut Lundquist

Christian Luschnig

Joseph Lynch

Hong Ma

Jian Feng Ma

Li-Jun Ma

Wei Ma

Gustavo C MacIntosh

Francisco Madueno

Takaki Maekawa

Steven Maere

Ari Pekka Mähönen

Alexis Maizel

Ian T. Major

Pal Maliga

Manuel Jesus Mallen-Ponce

Julin N. Maloof

Pablo Andrés Manavella

Nikolay Manavski

Concepcion Manzano

Chuanzao Mao

Ying-Bo Mao

David L Des Marais

Alexandre P. Marand

D. Blaine Marchant

L & Marechal-Drouard

Stéphane Mari

Hernan Dario Lopez Marin

Sebastian Marquardt

André Marques

Nayelli Marsch-Martínez

Antoine Martin

Cathie Martin

Guiomar Martín

Federico Martinez-Seidel

Daisuke Maruyama

Yoshikatsu Matsubayashi

Yusuke Matsuda

Kenji Matsui

Sachihiro Matsunaga

Makoto Matsuoka

Marjori Ann Matzke

Christophe Maurel

Jared May

Donald R. McCarty

Heather E. McFarlane

Karen McGinnis

Juliette de Meaux

Kunrong Mei

Vanessa J Melino

Charles Melnyk

Rainer Melzer

Siegbert Melzer

Dong Meng

Fanli Meng

Xiangzong Meng

Raphael Mercier

Rémy Merret

Carlos Messina

Etienne H Meyer

Yansong Miao

Mara Miculan

Ruben Milla

Ron Mittler

Kenji Miura

Takuya Miyakawa

Chikahiro Miyake

Shuhei Miyashita

Guillaume Moissiard

Ian Max Møller

Attila Molnar

Sébastien Mongrand

Isabel Monte

Bruna Montes-Luz

Beronda L. Montgomery

Laura Moody

Steve Moose

Santiago Mora-Garcia

John A Morgan

James V Moroney

Tomas Morosinotto

Panagiotis Nikolaou Moschou

Matthew James Moscou

Hiroyasu Motose

Gloria K. Muday

Sabine Mueller

Oliver Mueller-Cajar

Niels A Müller

Conrad W. Mullineaux

Paula Mulo

Manisha Munasinghe

Andrew Muroyama

Jeremy D Murray

Michael Muszynski

Alan M. Myers

Akira Nagatani

Peter Nagy

Masato Nakai

Keiji Nakajima

Masayoshi Nakamura

Yuki Nakamura

Masayoshi Nakayama

Eiji Nambara

Naweed Isaak Naqvi

Utpal Nath

Wojciech J Nawrocki

Andrew D. L. Nelson

Nathalie Nesi

Benjamin Neuhaeuser

Henry T. Nguyen

Thao Thi Nguyen

Zhongfu Ni

Jörg Nickelsen

Chad E Niederhuth

Janusz Niedojadlo

Erik Nielsen

Zoran Nikoloski

Lachezar A. Nikolov

Peter J. Nixon

Krishna K. Niyogi

Steve van Nocker

Michael Nodine

Maria A. Nohales

Mika Nomoto

Ken-Ichi Nonomura

Helen M. North

Trent Northen

Marc M Nowaczyk

Adriano Nunes-Nesi

Dmitri A. Nusinow

Laurent Nussaume

Richard O'Connell

Atsushi Oda

Remko Offringa

Uwe Ohler

Masaru Ohme-Takagi

Kenneth M. Olsen

Neil E. Olszewski

Diarmuid S. O'Maoileidigh

Joyce Onyenedum

Naomi Ori

Beatriz Orosa-Peunte

Anna Ostendorp

Lars Ostergaard

Artur Osyczka

Marisa S. Otegui

Xinhao Ouyang

Yidan Ouyang

Peggy Ozias-Akins

Gabriela C Pagnussat

Himadri B Pakrasi

Jose M. Pardo

Isobel Parkin

Harriet T Parsons

Stephen Pearce

Ales Pecinka

Ullas V Pedmale

Gilles Peltier

Steven Penfield

Mariano Perales

Imara Y. Perera

Staffan Persson

Paolo Pesaresi

Michael A. Phillips

Ronald Pierik

Craig S. Pikaard

Guillaume Pilot

Matthieu Pierre Platre

Bram Van de Poel

Brigitte Poppenberger

Charlotte Poschenrieder

Kalika Prasad

Gernot Presting

Jill Christine Preston

Daniil M Prigozhin

Holger Puchta

Michael D. Purugganan

Sujith Puthiyaveetil

Bao-xiu Qi

Qian Qian

Hong Qiao

Feng Qin

Yuan Qin

Yongjian Qiu

Leandro Quadrana

Volodymyr Radchuk

Sylvain Raffaele

Sunil Kumar Kenchanmane Raju

Aurelie Rakotondrafara

Aashish Ranjan

Aaron M Rashotte

Delfina A Re

Jason W. Reed

Haiyun Ren

Philippe Reymond

Ivan Reyna-Llorens

Jack Rhodes

Melanie Rich

Eric Jean Richards

Andreas S. Richter

Rosa M Rivero

Mercedes Rocafort

Jean-David Rochaix

Daniel Rodriguez-Leal

Rogelio Rodríguez-Sotres

Adrienne H. K. Roeder

Rob Roelfsema

Marcela Rojas-Pierce

Enrique Rojo

Filip Rolland

Hardy Rolletschek

Rebecca Roston

Hatem Rouached

Sonali Roy

Yong-Ling Ruan

Vicente Rubio

Sandrine Ruffel

Nils Rugen

Yue Rui

Javier Ruiz-Albert

Daniel Runcie

Wataru Sakamoto

Jorge E. Salazar-Henao

Marketa Samalova

M. Virginia Sanchez-Puerta

Diana Santelia

Julia Santiago

Ryoichi Sato

Eugene Savenkov

Ruairidh Sawers

Michael J. Scanlon

Gabriel Schaaf

Andreas Schaller

G. Eric Schaller

Hubert Schaller

David Scheuring

Katharina Schiessl

Jos H.M. Schippers

Eric A Schmelz

Robert J. Schmitz

Christian Schmitz-Linneweber

Kay Schneitz

Thorsten Schnurbusch

Mark Aurel Schöttler

Tina B. Schreier

Kathrin Schrick

Michael Schroda

Julian I Schroeder

Robert C. Schuurink

Claus Schwechheimer

Rajandeep S Sekhon

David Seung

Saima Shahid

John Shanklin

Gunjan Sharma

Lisha Shen

Hua Shi

Kai Shi

Xuetao Shi

Alexandra M. Shigenaga

Shahid Siddique

Lyudmila Sidorenko

Tobias Sieberer

Leslie E. Sieburth

Wendy K. Silk

Anne Simon

Jeffrey P Simpson

June K Simpson

R. Keith Slotkin

Ian D. Small

Alison G. Smith

Dariusz Jan Smolinski

Kimberley Cathryn Snowden

Roman Sobotka

Chun-Peng Song

Junqi Song

Rentao Song

Susheng Song

Xianjun Song

Pierre Sourdille

Erin E Sparks

Nese Sreenivasulu

Johannes Van Staden

Dorothee Staiger

Martin Stegmann

Adam D Steinbrenner

Mark Stitt

Sophia L Stone

Josh Strable

Dominique Van Der Straeten

Deserah D Strand

Daniela Strenkert

Robert M Stupar

Chao Su

Xiaodong Su

Sibum Sung

Ramanjulu Sunkar

Kankshita Swaminathan

Joseph Swift

Gwen Swinnen

Ildiko Szabo

Olga Sztatelman

Zofia Szweykowska-Kulinska

Daniel B. Szymanski

Koji Takahashi

Taku Takahashi

Yuichiro Takahashi

Kentaro Tamura

Lubin Tan

Ryouichi Tanaka

Wenqiang Tang

Christophe Tatout

Yuanwen Teng

Christa Testerink

Ian Joseph Tetlow

Steven M. Theg

Günter Theißen

Jay J. Thelen

Dhineshkumar Thiruppathi

Bart Thomma

Feng Tian

Zhixi Tian

Jurriaan Ton

Hongning Tong

Nadine Töpfer

Christopher N. Topp

Masatsugu Toyota

Kay Trafford

Marco Trujillo

Yu-Chang Tsai

Katsutoshi Tsuda

Kenichi Tsuda

Matthew R. Tucker

Franziska Katharina Turck

Simon R. Turner

Esa Tyystjarvi

Cristobal Uauy

Takashi Ueda

Richard Glen Uhrig

James G. Umen

Suayib Üstün

Henry E. Valentin

Marc Valls

Robert VanBuren

Klaas Vandepoele

Kranthi Varala

Imre Vass

Hervé Vaucheret

Jeanmarie Verchot

Stephane Verger

Joop Vermeer

Lieven De Veylder

Luis Vidali

Jean-Philippe Vielle-Calzada

Stefania Viola

Corina Vlot

Lars M. Voll

Elsbeth Lewis Walker

Ian S. Wallace

Justin W Walley

Aide Wang

Bing Wang

Guan-Feng Wang

Guo-Liang Wang

Kan Wang

Kehua Wang

Kejian Wang

Lei Wang

Shaokui Wang

Wen-Ming Wang

Xiaofeng Wang

Xiaojie Wang

Xuelu Wang

Ying Wang

Yingxiang Wang

Yong-Fei Wang

Yonghong Wang

Yu Wang

Yuichiro Watanabe

Mark Waters

Fabrice Wattebled

Andreas P.M. Weber

Yangdou Wei

Dolf Weijers

Bernd Weisshaar

Elina Welchen

Frank Wellmer

Ralf Welsch

Stephan Wenkel

Daniele Werck-Reichhart

James Whelan

Clinton Whipple

Charles I. White

Joshua R Widhalm

Emilie Wientjes

Klaas Jan van Wijk

Björn C. Willige

Zoe A. Wilson

Brenda S.J. Winkel

Tyler M. Wittkopp

Francis-Andre Wollman

Jesse D Woodson

Stephen Wright

Tadeusz Wroblewski

Michael Wrzaczek

Aimin Wu

Changyin Wu

Chaohua Wu

Keqiang Wu

Qingyu Wu

Shuang Wu

Ting Wu

Rui Xia

Daoquan Xiang

Yun Xiang

Fangming Xiao

Jun Xiao

Shunyuan Xiao

Yanjie Xie

Zhixin Xie

Yongzhong Xing

Lizhong Xiong

Changcheng Xu

Chunhui Xu

Dongqing Xu

Guixia Xu

Lin Xu

Qiang Xu

Shengbao Xu

Yong Xu

Chizuko Yamamuro

Takafumi Yamashino

Fei Yan

Shunping Yan

Shuichi Yanagisawa

Chengwei Yang

Dong-Lei Yang

Hong-Quan Yang

Jinliang Yang

Seong Wook Yang

Shuhua Yang

Wannian Yang

Xiaojing Yang

Xueyong Yang

Zefeng Yang

Zhenbiao Yang

Zhong-Nan Yang

Marcelo Javier Yanovsky

Levi Yant

Nan Yao

Ruifeng Yao

Keke Yi

Won Cheol Yim

Yanhai Yin

Chan Yul Yoo

Kaoru Okamoto Yoshiyama

Bin Yu

Jianming Yu

Jianping Yu

Qingyi Yu

Su-May Yu

Xiao Yu

Ling Yuan

Yao-Wu Yuan

Zheng Yuan

Fernando J Yuste-Lisbona

Viktor Zarsky

Raul Zavaliev

Jürgen Zeier

Yonglun Zeng

William Zerges

Jixian Zhai

Junpeng Zhan

Li Zhang

Qun Zhang

Shuqun Zhang

Weiwei Zhang

Wenhua Zhang

Xian Sheng Zhang

Xianlong Zhang

Xiaolan Zhang

Xing Zhang

Yan Zhang

Yinwen Zhang

Yongliang Zhang

Zhengguang Zhang

Dazhong Dave Zhao

Meixia Zhao

Yang Zhao

Binglian Zheng

Shangwei Zhong

Silin Zhong

Hai Zhou

Jian-Min Zhou

Jie Zhou

Mian Zhou

Pei Zhou

Yuyi Zhou

Mingyuan Zhu

Xiaohong Zhuang

Chloë Zubieta

## NEW REVIEWING EDITORS IN 2023

Marc Somssich

Shuhua Yang

## NEW GUEST EDITORS IN 2023

Gabriela Auge

Lieven De Veylder

Candy Hirsch

Tomokazu Kawashima

Ullas Pedmale

Li-Jia Qu

Sonali Roy

Yong-Lin Ruan

Sebastian Soyk

Sébastien Thomine

Cristobal Uauy

Shuang Wu

## OUTGOING EDITORS IN 2023

Sean Cutler

Zach Lippman

Terri Long

Beronda Montgomery

Michael Udvardi

Dabing Zhang

